# Medication Adherence Measures: An Overview

**DOI:** 10.1155/2015/217047

**Published:** 2015-10-11

**Authors:** Wai Yin Lam, Paula Fresco

**Affiliations:** ^1^UCL School of Pharmacy, 29-39 Brunswick Square, London WC1N 1AX, UK; ^2^Department of Drug Sciences, Laboratory of Pharmacology, Faculty of Pharmacy, University of Porto, Rua Jorge Viterbo Ferreira 228, 4050-313 Porto, Portugal

## Abstract

WHO reported that adherence among patients with chronic diseases averages only 50% in developed countries. This is recognized as a significant public health issue, since medication nonadherence leads to poor health outcomes and increased healthcare costs. Improving medication adherence is, therefore, crucial and revealed on many studies, suggesting interventions can improve medication adherence. One significant aspect of the strategies to improve medication adherence is to understand its magnitude. However, there is a lack of general guidance for researchers and healthcare professionals to choose the appropriate tools that can explore the extent of medication adherence and the reasons behind this problem in order to orchestrate subsequent interventions. This paper reviews both subjective and objective medication adherence measures, including direct measures, those involving secondary database analysis, electronic medication packaging (EMP) devices, pill count, and clinician assessments and self-report. Subjective measures generally provide explanations for patient's nonadherence whereas objective measures contribute to a more precise record of patient's medication-taking behavior. While choosing a suitable approach, researchers and healthcare professionals should balance the reliability and practicality, especially cost effectiveness, for their purpose. Meanwhile, because a perfect measure does not exist, a multimeasure approach seems to be the best solution currently.

## 1. Introduction

Adherence to medication is a crucial part of patient care and indispensable for reaching clinical goals. The WHO, in its 2003 report on medication adherence, states that “increasing the effectiveness of adherence interventions may have a far greater impact on the health of the population than any improvement in specific medical treatment” [1]. By opposition, nonadherence leads to poor clinical outcomes, increase in morbidity and death rates, and unnecessary healthcare expenditure [2, 3]. While noncommunicable and mental illnesses are expected to exceed 65% of the global burden of disease in 2020 [1], approximately 50%–60% of patients are nonadherent to the medicine that they have been prescribed, especially those suffering from chronic diseases [4, 5]. As a result, more than 30% of medicine-related hospital admissions occur due to medication nonadherence [6, 7].

The WHO defines* adherence* as “the extent to which the persons' behavior (including medication-taking) corresponds with agreed recommendations from a healthcare provider” [1]. It includes the* initiation* of the treatment,* implementation* of the prescribed regime, and* discontinuation* of the pharmacotherapy [8]. Meanwhile, some studies classify adherence as either primary or secondary. Primary nonadherence is the frequency with which patients fail to fill prescriptions when new medications are started so it is related to refilling and initiation of the medication therapy [9]. Secondary nonadherence is defined as the medication being not taken as prescribed when prescriptions are filled. It does not only affect the clinical outcome but also affect the financial outcome of the health system [10].

Often, compliance is also used and the two can be used interchangeably in research and clinical practice [11]. It describes “the extent to which the patients' behavior (including medication-taking) coincides with medical or healthcare advice” [12], yet its meaning has become more negative regarding patient's behaviors, since it implies patient's passivity [13]. Therefore, in this paper,* adherence* will mainly be used. However, according to Steiner and Earnest, both terms present problems as to describe medication-taking behaviors since they inflate the physician's control over the medication process [14].

Poor medication adherence has multifactorial causes that need to be understood before interventions can be designed to improve medication adherence [2]. According to WHO, there are multiple factors leading to poor medication adherence, normally classified into five categories: socioeconomic factors, therapy-related factors, patients-related factors, condition-related factors, and health system/health care team- (HCT-) related factors [1]. With an understanding of whether the nonadherence is primary (initiation of pharmacotherapy) or secondary (implementation of the prescribed regime), and what factors have led to it, a proper intervention can then be tailored individually to improve the medication-taking behavior of each patient.

Measuring adherence is, therefore, important to both researchers and clinicians. Inaccurate estimation of medication adherence can lead to several problems which are potentially costly and dangerous in both settings. Effective treatments may be judged as ineffective, expensive diagnostic procedures may be ordered, and therapy may be unnecessary and dangerously intensified. In addition, results of clinical trials cannot be realistically interpreted without adherence information [15, 16]: treatment efficacy and dose-response relationships are miscalculated in studies where patients present poor adherence. Moreover, accurate estimates of medication adherence will provide better evidence on the consequences, predictors/risk factors, and strategies to improve medication adherence.

Nevertheless, measurement of medication adherence can be quite challenging since and parameters of acceptable adherence need to be carefully delineated and appropriated for individual situations [17]. There are numerous tools available for these measurements, but these need to prove to be valid, reliable, and sensitive to change [13, 17]. The selection of a method to monitor adherence should be based on individual attributes and goals/resources of the study or the clinical setting. Currently none of the available methods can be considered as a gold standard and the combination of methods is recommended [15].

However, even after decades of research, there is very little guidance for healthcare professionals and researchers to choose the most suitable adherence measures. The aim of this paper is to give an overview of validated and commonly used medication adherence measures and a general scope for identifying nonadherence in common situations.

## 2. Overview

For more than four decades, numerous researches on how to properly measure and quantify medication adherence have been conducted but none of them can be counted as the gold standard. Different tools have been designed and validated for different conditions, in different circumstances. Generally, measurements of medication adherence are categorized by the WHO as subjective and objective measurements [1].

Subjective measurements involve those requiring provider's or patient's evaluation of their medication-taking behavior. Self-report and healthcare professional assessments are the most common tools used to rate adherence to medication [18]. The most common drawback is that patients tend to underreport nonadherence to avoid disapproval from their healthcare providers [19].

Objective measures include pill counts, electronic monitoring, secondary database analysis and biochemical measures and are thought to represent an improvement over subjective measures [13, 18]. As such, objective measures should be used to validate and correlate the subjective ones. However, a meta-analysis on adherence outcomes states that a multi-subjective-measure approach may have higher sensitivity, but not accuracy, over employing a single objective measure [20]. In summary, subjective and objective measures have both advantages and disadvantages and should be used in combination. Further details will be individually discussed below.

## 3. Direct Measures

In addition to the classification of adherence measures as subjective and objective, many other studies labeled them as* direct* and* indirect* [7, 15, 21, 22]. Direct measures include measurement of the drug or its metabolite concentration in body fluids, such as blood or urine and evaluation of the presence of a biological marker given with the drug and direct observation of patient's medication-taking behavior. These measures can be made randomly or at specific intervals [15].

Even though direct measures are considered to be the most accurate and can be used as a physical evidence to prove that the patient has taken the medication, there are many drawbacks regarding their use. They simply generate a Yes/No result without revealing any pattern of the nonadherence or their causes [15]. Tests themselves can also be very intrusive which may cause pressure and anxiety in patients.

Drug metabolism should be taken into account while considering using these methods. For instance, traces of neuroleptic and psychiatric medications can be detected in the blood even long after stopping the medication. Since individuals vary in physiological state and metabolic rate, drug plasma levels also differ after different individuals take the same dose of the same medicine. Moreover, the quantification itself can be difficult. For instance riboflavin, a biological marker, is simply nonquantitative for detection [23]. Additionally, drug-drug interactions and drug-food interactions can hinder the assay's accuracy. Therefore, these direct methods are generally unsuitable for psychiatric patients and those under multidrug regimes, even when they are hospitalized.

Furthermore, direct measures are very expensive and difficult to perform as many technicians and professionals are required to monitor the process and carry out the tests. Using direct observation as an example, patients can hide their medicines under tongue and discard them afterwards, making routine inspection impractical. Therefore, these measures are mostly used for patients under single-dose therapy or intermittent administration and hospitalized [13].

Bias can also be introduced if patients take the medication only before the upcoming tests. White coat adherence [7, 22] is a phenomenon that cannot be ignored in any study involving direct measures or visits from healthcare professionals. It is normally described as the “improved patient adherence to treatment around clinic visits” [24, 25]. Modi et al. reported an average of 88% and 86% adherence rates before and after the visit, respectively, but adherence rates declined to 67% a month after the visit [25]. This suggests that a false adherence may occur and should be considered while carrying these measures. Ideally, healthcare professionals should not inform patients of the visit's date to minimize this barrier, yet it challenges the right of patients to control their own treatment [26].

## 4. Measures Involving Secondary Database Analysis

The data of secondary database includes the sequences and patterns derived from the curated primary data in systems such as electronic prescription service or pharmacy insurance claim. Such data allows quantification of medication adherence to various refill adherence measures. Refill adherence assumes that prescription-refilling patterns correspond to the patient medication-taking behavior. This assumption has been considered as an acceptable estimate [29]. Furthermore, these measures also assume that the medication is taken exactly as prescribed [27]. As a result, partial adherence where patients only take a part of the medication in that interval cannot be revealed using these measures.

Farmer has divided refill adherence into 3 types: continuous variable, which is assessed “from the first to the last prescription record,” such as the Medication Possession Ratio (MPR);* dichotomous variable*, in which “patients are categorized as either compliant or noncompliant based on criteria such as a specified treatment gap”; and examining “the time between prescription refills from the perspective of time gaps (periods of nonadherence) or consumption (medication availability, the days' supply/days between refills),” for example, Continuous Measure of Medication Acquisition (CMA), Continuous Measure of Medication Gaps (CMG), Continuous, Single Interval Measure of Medication Acquisition (CSA), and Continuous, Single Interval Measure of Medication Gaps (CSG)[15]. Many studies using secondary database analysis also utilize Proportion of Days Covered (PDC). However, this tool is a measure of persistence to the medication therapy, instead of adherence.

Reviewing prescription refill records requires a centralized computerized system along with a consistency among prescribers and dispensers to collect a complete dataset over that designated period [15]. This allows an analysis of a large population and results in the popularity of this method in research. Moreover, this method is able to assess multidrug adherence and to identify patients at risk for treatment failure [30]. Even though barriers, such as demographic features, can be compared and pinpointed as nonadherence factors, this method does not give many clues to the researcher or the health professionals concerning the barriers involved in the detected nonadherence in terms of individual patient [31].

To avoid errors from inaccurate data input, administrative datasets compiling billing information for healthcare service [28] and insurance claims are often used in research, as this complete dataset, including all prescription activities, is verified by insurance companies or prescription benefit managers (PBMs) in the United States [15]. The authors stated that researchers should bear in mind that they must be able to verify the continuous patient's eligibility to participate and to “differentiate treatment cessation from patient death or switches in insurance plans.” Furthermore, researchers should be aware that some prescriptions may be missed out if they are obtained outside of the insurance plan as well as any drug discontinuation advised by prescriber verbally, without record [27]. Therefore, utilizing the database for refill adherence is intended for consistent, nondiscretionary use.  Table 1 presents the equation of each method described below.

### 4.1. Medication Possession Ratio (MPR)

Andrade et al. defined MPR as the proportion (or percentage) of days' supply obtained over either* refill interval*, where last refill is the end point, or* fixed* refill, where a specific time period is set [32]. The former is used in the case such as patients with depression or HIV whereas the latter is generally used for assessing seasonal use of medication, asthma or allergies, and so forth [29]. The denominator variation makes MPR impossible to use on a large population analysis. Hence, appropriate correlation and average would be necessary to adjust for overall adherence values [28]. It is a very simple calculation method which does not consider the gaps in refills and “the need for continuous therapy with multiple prescriptions” [33]. Consequently, overestimated adherence values are found while using this method.

### 4.2. Dichotomous Variable

This measure requires a cutoff value to distinguish adherence and nonadherence or adherence from partial adherence [15, 27]. Compared to the continuous variable, it has lower sensitivity probably due to its general lack of pharmacological basis for deciding the cutoff value [15]. This greatly affects the sensitivity and specificity of the test's results. These drawbacks made some authors to recommend the use of continuous variable measures instead, since they show higher reliability and power [34].

### 4.3. Continuous, Multiple Interval Measure of Medication Acquisition (CMA)

CMA is calculated as the cumulative days' supply obtained over a series of intervals divided by the total days from the beginning to the end of the time period in study. The overall average of all participants' CMA provides the adherence value of the entire time period of the study [28] and evaluates the relationship of adherence and drug effect [27]. Hess et al. suggest that CMA and MPR, along with Continuous Multiple Interval Measure of Oversupply (CMOS) and Medication Refill Adherence (MRA), provide identical adherence measuring power [28].

### 4.4. Continuous, Multiple Interval Measure of Medication Gaps (CMG)

CMG measures are obtained dividing the total number of days in treatment gaps by the duration of the time period of interest in order to recognize any time intervals without drug exposure [27]. Any negative value would be set to 0. It calculates nonadherence values for cumulative periods without considering the possibility of early refill or overfill. If any surplus is included, CMOS should be used to adjust for oversupplies obtained during earlier prescription intervals to incorporate any excess medication within the time period [28].

### 4.5. Continuous, Single Interval Measure of Medication Acquisition (CSA)

CSA is determined by the days' supply obtained in each interval over the total days in the interval [27]. Bias occurs when the patient gets more than one refill a day or when refill is close to the day of completion [28].

### 4.6. Continuous, Single Interval Measure of Medication Gaps (CSG)

CSG identifies time periods during which medication exposure is unlikely. It is calculated by the number of days without any medication over number of days in the interval. Similar to CSA, CSG is more suitable for short-term drug exposure, such as the patients with only one prescription and the short-term drug usage is related to clinical outcome [27].

## 5. Measures Involving Electronic Medication Packaging (EMP) Devices

EMP devices are “adherence-monitoring devices incorporated into the packaging of a prescription medication.” With several choices available, they share some common features: (i) recorded dosing events and stored records of adherence; (ii) audiovisual reminders to signal time for the next dose; (iii) digital displays; (iv) real-time monitoring; and (v) feedback on adherence performance [35]. The popularity of above features that appear in devices is ranked in descending order. Even though not all such features are available in all devices, recording adherence performance is essential for analysis and to tailor suitable interventions. The Medication Events Monitoring System (MEMS) is the most commonly used EMP device in medication adherence studies.

### 5.1. Medication Events Monitoring System (MEMS)

Even though various models have been designed over decades, the basic principle of this system is that whenever the medication is removed from the container, a microprocessor embedded would record the time and date, assuming that the patient has taken that specific dose at that particular time [5, 15, 23].

This objective measure is being highly accurate in several studies [5]. It helps identify whether the nonadherence is sporadic or consistent or any other abnormal medication-taking pattern and it is able to detail the number of daily doses on any partial adherence situation. These features make MEMS more useful than biochemical and self-report measures [15]. Additionally, the tendency of deceiving is lower than when using pill count as the patient needs to open the container every day at the same time if they want to discard the medication to guarantee that the same “adherence” pattern is recorded [23]. As a result, it is always used as a reference standard for validating other adherence measures.

Despite the fact that more effort is needed to create the false impression of adherence, there is no assurance that patient would not do it. Apart from purposefully misleading the system, patients may accidentally actuate the container without taking the medication [15]. This can lead to medication adherence overestimation.

The bulkiness of the container is also an obstacle, which can make patients transfer the medication into another container or not carry the medication when they go out [15, 23]. Furthermore, the presence of the container alone may keep reminding the patient that they are under surveillance. This has been reported to result in anxiety, stress, and somatic complaints in some cases [15].

Although the accuracy of MEMS is undeniable, its lack of interest for studies with large populations, such as clinical trials, or routine use is related to high costs and the amount of support required [5, 15, 23, 35]. The equipment alone is very expensive. With the possibility of equipment loss by patient, rental of hardware and software for data retrieval, staff time, bed days, and the cost to encourage patients to return the cap, MEMS studies require large funds to complete. A total of USD$274 per patient was required to complete a 6-month study in a 2001 study that estimated medication adherence in patients with schizophrenia or schizoaffective disorder [23]. These authors also mentioned other practical issues, including the difficulty in coordinating refills with outpatient pharmacies and the need to encourage patients for the correct use of the cap [23]. The incorrect use of the MEMS container may lead to false categorization of patients as nonadherent [36].

## 6. Pill Count

This indirect, objective measure counts the number of dosage units that have been taken between two scheduled appointments or clinic visits. This number would then be compared with the total number of units received by the patient to calculate the adherence ratio [15, 19].  Table 1 presents the equation based on the definition. The low cost and simplicity of this method contribute to its popularity. However, several limitations have been identified.

First, although it can be used for various formulations, such as tablets, capsules, and actuated inhaler, this approach is unfeasible in assessing those with nondiscrete dosages or* Pro re nata* (prn) medication [19].

Moreover, adherence underestimation occurs frequently, since this method simply uses the dispensed date as the denominator of the equation without considering the chance of having surplus medication. Especially for patients with chronic conditions, it is common for them to refill the medication before running out [19]. Moreover, the cutoff value to differentiate adherence and nonadherence, in this case, is generated arbitrarily [15]. This can lead to discrepancy on determining patient's adherence and comparing medication adherence among studies.

Although pill count is based on a similar assumption to MEMS, which is the fact that the removal of the dosage unit is equivalent to taking the medication, pill count does not generate a medication-taking pattern as the latter does. Removing the correct number of dosage units from the container does not necessarily mean the patient follows the dosing regime consistently [36, 37]. Besides pill count's inability to characterize the adherence pattern, it is also unable to identify its causes [15].

Pill count has shown higher accuracy comparatively to other subjective methods, but MEMS has replaced pill count as a reference standard for validating other adherence measures in the 1990's [15].

## 7. Measures Involving Clinician Assessments and Self-Report

Many authors believe that these subjective methods are the least reliable among all. Nevertheless, their low cost, simplicity, and real-time feedback have contributed to their popularity in clinical practice [4, 38, 39]. They can be administered as structured interviews, online assessments, written questionnaires, voice response system, and so forth. Additionally, due to their practicality and flexibility, these questionnaires are able to identify individual patient concerns and subsequently tailor appropriate intervention [5].

Surely, the drawbacks of such approaches should not be undermined. The relatively poor sensitivity and specificity can occur due to false data input by patients, purposefully or accidently [21, 38], or faulty communication skills and questions constructed by the interviewers as well as the design of survey [15, 23]. Negativity in questions, suggesting blaming the patients for not fulfilling their prescribed regime, may lead to bias [15]. Patient's psychological state can also impact the response [5]. As a result, such objective measures can only weakly predict patient's adherence and are more commonly used in clinical practice than research.

### 7.1. Patient-Kept Diaries

This is the only self-report tool that is consistently documented with how the patient follows their prescribed regime. However, overestimation is very common and an average of 30% surplus of diary entries has been shown to occur when comparing with different results from MEMS data [15]. Authors also mentioned other factors that can contribute to its unreliability, including the inability to carry out the assessment if the patient does not return the diary or the reported “false” increase in patient's adherence rate from monitoring phase to self-assessment phase [40].

### 7.2. Patient Interviews

Interviewing patients by clinicians is generally an easy-to-use, low-cost subjective method to assess patient's adherence [15]. Patients can be asked to estimate their own medication-taking behavior, namely, which percentage of dose that they may miss within a designated period or the frequency that they are unable to follow the medication regime. Alternatively, questions can also be based on patient's knowledge on the personal prescribed regime, including drugs' name, schedule, and indications. Healthcare professionals then evaluate their response to determine the level of adherence. However, the authors also stated that there is only limited evidence on the relationship between the patient's knowledge on their medication regime and actual adherence [19].

Apart from the traditional approach described above, motivational interview has an increased popularity in clinical practice. This combines the adherence measuring and subsequent intervention into one tool. It does not only measure and evaluate medication adherence, but it intervenes if there is any case of medication nonadherence. Miller and Rollnick defined it as a direct patient-centered approach to help patients understand and resolve ambivalence so as to encourage behavioral changes [41]. Initially designed to combat substance abuse, it is aimed at identifying patient's resistance to change and motivating them via advice and questioning [42]. In a meta-analysis, Rubak et al. indicated that by its ability to combine identification of the causes behind nonadherence and subsequent intervention, motivational interviewing outperforms the traditional advice giving [43].

### 7.3. Questionnaires and Scales

These subjective approaches were first designed to minimize the limitations of other self-report methods by standardizing the measurement of adherence to a specific medication regime [15]. These questionnaires are generally validated against other measures, both subjective and objective, and with numerous versions to accommodate various conditions, such as for a broad-ranged or single diseased population, or in different languages. Self-report questionnaires should be completed by patients themselves or their caretakers. However, questionnaires can be difficult for patients with low literacy levels [44].

In a systematic review, Nguyen et al. have identified 43 validated self-report adherence scales, excluding those that were not in English [38]. 40 out of these 43 scales have weighed the extent of implementation of a dosing regime, including the initiation, implementation, and discontinuation phases. Furthermore, the authors categorized the scales into 5 main groups that evaluate the following: (i) only medication-taking behaviors; (ii) both medication-taking behavior and barriers to adherence; (iii) only barriers to adherence; (iv) only beliefs associated with medication adherence; and (v) both barriers to and beliefs associated with adherence. This review defined medication-taking behaviors as any missing dose taken, as well as frequency on prescription refill while barriers to adherence were defined as tendency to forget, disease-specific reasons, regime complexity, and/or side effect of prescribed medications. Beliefs associated with adherence are related to personal concerns on the medication safety or the need of following the prescribed regime.

In terms of determining nonadherence, these authors summarized the methodology for those scales. Most analyzed scales have a recommended cutoff value. Patients that took 80% or more of their medicines, as ascertained by an objective measure, for example, MEMS, are reported as adherent, and those who took less than this cutoff value are reported as nonadherent, whilst some may correspond to other self-report measures that had been accredited by objective measures in advance. Apart from correlation with other measures, the comparison of the adherence scale's mean scores of adherent and nonadherent populations can determine the cutoff value.

Meanwhile, some scales, like the Medication Adherence Questionnaire (MAQ), the 8-item Morisky Medication Adherence Scale (MMAS), and the Brief Medication Questionnaire, rank the degree of adherence instead of defining an absolute cutoff for adherence. The rationale of ranking can either be determined by clinical outcomes or researcher's expertise.

As many scales were identified, this paper will focus on those that are considered as the most useful covering the concept of medication-taking behaviors, barriers to adherence, and belief associated with adherence.

#### 7.3.1. Brief Medication Questionnaire

(The Brief Medication Questionnaire is not abbreviated as BMQ, since BMQ usually stands for Belief about Medicines Questionnaire.) The Brief Medication Questionnaire explores both patient's medication-taking behavior and barriers to adherence. It consists of three different screens, a 5-item* Regime* screen, a 2-item* Belief* screen, and a 2-item* Recall* screen. These screens assess how patients took each of their medications in the past week, on drug efficacy and bothersome features and remembering difficulties, respectively. Svarstad et al. further reviewed that, with its ability to allow self-administration, evaluate multidrug regimens, and reduce practitioner's training, this questionnaire is popular among healthcare professionals [5].

It has been first suggested for diabetes and depression management and, ideally, patient's prescribed regime should be reviewed before being administered. Thus, the entire process may be more time-consuming comparatively to other questionnaires, which makes it difficult to be scored at the point of care [4].

#### 7.3.2. Hill-Bone Compliance Scale (Hill-Bone)

As a measure of reviewing patient's medication-taking behavior and barriers to adherence, Hill-Bone has a limited generalizability since it targets patients with antihypertensive medication only. The test consists of 3 subscales, medication-taking behavior, ability to keep appointment, and sodium intake, and is rated on a four-point Likert-type scale. The number of items available for testing varies among population types. 14-item and 9-item tests have been validated for urban black and community-dwelling populations, respectively [4].

When first designed, it has showed high internal consistency [45] and so it did when used in a primary healthcare setting from a study in South Africa [46]. The authors also described that Hill-Bone has a higher performance for black than nonblack populations despite its high cultural sensitivity [47]. Meanwhile, the study with community-dwelling population also proved its high internal consistency in outpatient settings [48]. Therefore, this scale has been suggested as suitable for use in studies specific for hypertension in a predominantly black population.

#### 7.3.3. Eight-Item Morisky Medication Adherence Scale (MMAS-8)

Based on the MAQ, Morisky et al. developed this 8-item MMAS (MMAS-8) in 2008. The first seven items are Yes/No responses while the last item is a 5-point Likert response. The additional items focus on medication-taking behaviors, especially related to underuse, such as forgetfulness, so barriers to adherence can be identified more clearly [44].

93% sensitivity and 53% specificity were reported while validating in “very low income minority patients treated for hypertension seeking routine care in a clinic setting” [39]. MMAS was also validated with outstanding validity and reliability in patients with other chronic diseases [44]. As a result, it is probably the most accepted self-report measure for adherence to medication.

Along with blood pressure control data, MMAS should be able to identify medication nonadherence and help control blood pressure [39]. Therefore, it is recommended to serve as a screening tool for validated conditions in the clinic setting.

#### 7.3.4. Medication Adherence Questionnaire (MAQ)

The MAQ is also known as the 4-item Morisky Medication Adherence Scale (MMAS-4) and Morisky Scale [4, 38, 44, 49]. This questionnaire is the quickest to administer and score and is only able to identify barriers to adherence due to its length [4]. The closed question format with “yes-saying” bias allows disclosures of nonadherence [44]. Since it has been validated in the broadest range of diseases and in patients with low literacy, it is the most widely used scale for research [49].

In a study on factor structure and validity of MAQ for cigarette smokers, it was reported that the coefficient alpha reliability of MAQ varied among studies as well as validity estimates [50]. Compared to MMAS-8, MAQ has poorer psychometric properties. In the first validation for hypertensive population, the sensitivity and specificity were 81% and 44%, respectively [51]. As a result, MMAS-8 has become more popular than MAQ.

#### 7.3.5. The Self-Efficacy for Appropriate Medication Use Scale (SEAMS)

The SEAMS is a 13-item, 3-point Likert-type scale focusing on self-efficacy in chronic disease management while measuring barriers to medication adherence. It may be difficult to carry out at the point of care because of its length. However, this scale has been validated in various chronic conditions [4, 49].

Reliability of this scale was measured by its internal consistency. With coefficient alpha reliability at 0.89 and 0.88, on low and high literacy populations, respectively [4], SEAMS is, therefore, considered as an excellent self-report tool for measuring medication adherence in chronic diseases management.

#### 7.3.6. Medication Adherence Report Scale (MARS)

MARS assesses both beliefs and barriers to medication adherence [38]. It is based on the Drug Attitude Inventory (DAI), a common psychiatric adherence survey. By incorporating the questions from MAQ, it aims to reduce the deficiencies of DAI. As a result, it is able to examine medication-taking behaviors and attitudes toward medication with higher validity and reliability values. It consists of 10 questions with a simple scoring to evaluate patient's adherence behaviour, attitude towards medication, and general disease control during the past week [52].

The internal consistency reliability of MARS is unclear [4]. Still, Thompson et al. showed that this scale has strong positive correlations compared to DAI and MAQ. It was designed and first validated for patients with schizophrenia [52]. Hence, this scale is limited to use in patients with chronic mental illness.

## 8. Choosing a Suitable Medication Adherence Measure

An ideal medication adherence measure should present low cost and be user friendly, easy to carry out, highly reliable, flexible, and practical [13, 15]. However, there is no single measure that can meet all these gold standards since each has its own drawbacks as described above.

In a broad sense, subjective and objective measures are preferred in clinical and research settings, respectively, mainly due to cost effectiveness ratios. Self-report questionnaires, which have a reasonable predictive power, are more useful in a busy, resource-limited clinical setting with moderate to high literacy population. Patient's interview by clinicians is preferred for low literacy population or acts as an adjunct where patients have already been predicted as low medication adherers. Although pill count is an objective measure, the needs of staff and time have made it primarily used in routine clinical practice instead. While balancing accuracy and cost, pharmacy refill measures are more favorable for a large research population than using EMPs. Meanwhile, direct measures are seldom used since the intrusiveness and the cost are too high to be accepted by both patients and researchers.  Table 2 includes advantages, disadvantages, and the proposed target population(s) of the five types of medication adherence measures whilst Table 3 summarizes the function(s), target population(s), advantages, and disadvantage(s) specific to the discussed self-report questionnaires and scales.

## 9. Multimeasure Approach

Multimeasure approach is often recommended in measuring medication adherence. Since there is no ideal medication adherence measure, it is appropriate to use more than one measure when researchers intend to have results that are close to reality. Selecting two (or more) medication adherence measures might allow strengths of one method to help compensate putative weakness and to more accurately capture the information needed to determine adherence levels. A study using this approach which measured the adherence to HIV protease inhibitors in 2001, Liu et al. showed that the composite use of MEMS, pill count, and clinician's interview held the strongest predictive power compared to the power when each measure was used separately [53].

An individual tool can only detect patients with low to moderate level of adherence. Other factors, such as white coat adherence, can lead to a false impression of medication adherence. The use of a second measure can then help confirm the original findings. For instance, although MEMS is known for its high accuracy, adherence overestimation may still occur when using this method. Therefore, some studies use other measures in addition to MEMS, such as pill count, to attest the result and minimize discrepancies [36, 37].

Moreover, different measure can identify different components of nonadherence. Subjective measures are more useful in determining the beliefs and barriers to adherence or predicting nonadherence. Objective measures provide more accurate data on how patients perform in their medication regimes. A simple self-report survey has been used to predict the occurrence of low pharmacy refills in a high-risk elderly population to improve hypertensive management [47]. A meta-analysis also showed that this approach, including using a self-report method other than medical record reviews alone, can increase the sensitivity for nonadherence [54]. The concomitant use of both objective and subjective measures will, therefore, provide higher reliability and reveal more reasons of nonadherence, even in patients with high levels of adherence, and is currently recommended [55].

Nonetheless, increased complexity for analysis and interpretation should be acknowledged when using a multimeasure approach, such as different timeframes for measurements and different results produced [56]. Meanwhile, using multiple measures with the same sources of error, such as two subjective measures, does not help predict adherence level [57]. The cost and practicality of this approach in clinical setting may also be a hindrance. Therefore, while choosing which measures should be included, researchers should take potential errors, ability to overcome the precedent disadvantages, and practicality to be performed in the target population into consideration.

## 10. Limitations

This is not a comprehensive review on all the existent medication adherence measures. Rather it is focused on the different types available and the most commonly used in different settings. The types of setting and population in the studies that are used as examples vary in different measures which can make comparisons cumbersome. If researchers and healthcare professionals are looking for measures for a specific or rare condition, they should refer to studies that have a clearer validation. Moreover, this review is limited to researchers and health professionals conducting studies in English language. Many measures have been translated and validated in several languages over the years of development yet this review does not include them.

## 11. Implications and Directions for Future Research

There are worldwide ongoing public health reforms to minimize unnecessary healthcare expenditure and maximize public health outcome. Improving medication adherence is a significant aspect in clinical practice and research. The lack of a universal guideline on medication adherence measures provides rooms for research on which measure, or which combination of measures, is the most appropriate for different target populations and health problems. Meanwhile, researches on improving the currently available measures and/or on the development of new ways to measure and uncover reasons behind medication nonadherence should also be further explored.

## 12. Conclusion

Poor medication adherence is a key hindrance in combating the challenges of public health in both developed and developing countries. For successful pharmacotherapy, healthcare professionals and researchers should utilize all available methods within their limits of practice to improve medication adherence. This study should be able to provide a general direction for professionals to choose the most suitable measures for their aims and subsequently deliver efficient, tailored interventions to improve patient's medication-taking behaviors.

## Figures and Tables

**Table 1:** Equations of medication adherence measures involving secondary database analysis and pill count [15, 19, 27, 28].

Measures	Equation
Medication Possession Ratio (MPR)	Days' supply obtained/refill interval or fixed interval

Dichotomous variable	N/A (arbitrary cutoff value)

Continuous, Multiple Interval Measure of Medication Acquisition (CMA)	Cumulative days' supply obtained over a series of intervals/total days from the beginning to the end of the time period

Continuous, Multiple Interval Measure of Medication Gaps (CMG)	Cumulative days without any medication over a series of intervals/total days from the beginning to the end of the time period

Continuous, Single Interval Measure of Medication Acquisition (CSA)	Days' supply obtained in each interval/total days in the interval

Continuous, Single Interval Measure of Medication Gaps (CSG)	Number of days without any medication/total days in the interval

Pill count	(Number of dosage units dispensed − number of dosage units remained)/(prescribed number of dosage unit per day × number of days between 2 visits)

**Table 2:** Summary of the five types of medication adherence measure: target population(s), advantages, and disadvantages.

Measures	Target population(s)	For primary/secondary nonadherence	Advantages	Disadvantages
Direct measures	Patients under single-dose therapy and intermittent administration and who are hospitalized	Both primary and secondary nonadherence	Most accurate Can provide physical evidence	Generate a Yes/No result only Intrusive Varied drug metabolism Nonquantifiable biomarkers/drug metabolites Drug-drug interactions and drug-food interactions Expensive Require qualified staff and techniques to perform Bias occurs if patients know the schedule of the tests (white coat adherence)

Measures involving secondary database analysis	Countries that allow refilling prescription; with centralized computerized system with a consistency among prescribers and dispensers; more common for research with a large population	Primary nonadherence	Able to assess multidrug adherence Can identify patients at risk for treatment failure Provide medication-refilling pattern Complete dataset used are generally verified by a third party for insurance claim purpose	Assumptions are made (the medication-taking behavior corresponds to prescription refilling and the medications are taken according to prescription) Fail to identify partial adherence Fail to identify barriers for the detected nonadherence Missing out prescriptions, if obtained outside the system Incomplete records, if drug discontinuation is verbally advised by prescriber

Measures involving Electronic Medication Packaging (EMP) devices	Studies with small population As reference standard to validate other measures	Secondary nonadherence	Highly accurate Identify medication-taking pattern Identify partial adherence	Expensive Technical supports required Overestimation if patients accidently or purposefully actuate the container Inconvenience due to bulky container Pressure to patients

Pill count	Routine clinical practice	Primary nonadherence	Low cost Simple Can be used in various formulations Highly accurate	Not for nondiscrete dosages or *prn* medications Underestimation due to early refill Arbitrary cutoff value Unable to identify medication-taking pattern

Measures involving clinician assessments and self-report	Routine clinical practice Less suitable for research	It depends on the type of assessments and questionnaires used	Low cost Easy to administer Real-time feedback Available Flexible to accommodate different conditions Identify belief and barriers to adherence Well-validated	Least reliable Relatively poor sensitivity and specificity Affected by communication skills of interviewers and questions in the questionnaire Patient's desirability can bias

**Table 3:** Summary of self-report questionnaire and scales: function(s), target population(s), advantages, and disadvantages.

Questionnaire and scales	Function(s)	Target population(s)	Advantages	Disadvantage(s)
Brief Medication Questionnaire	Patient's medication-taking behavior Barriers to adherence	Diabetes Depression	Self-administration Evaluate multidrug regimes Reduce practitioner's training	Time-consuming

Hill-Bone Compliance Scale (Hill-Bone)	Patient's medication-taking behavior Barriers to adherence	Hypertension specific, black patients	High internal consistency in both primary and outpatient setting	Limited generalizability

8-item Morisky Medication Adherence Scale (MMAS-8)	Patient's medication-taking behavior Barriers to adherence	All validated conditions	Higher validity and reliability in patients with chronic diseases than MAQ	

Medication Adherence Questionnaire (MAQ)	Barriers to adherence	All validated conditions	Quickest to administer Validated in the broadest range of diseases Validated in patients with low literacy	Comparatively short, mainly suitable for initial screening

The Self-Efficacy for Appropriate Medication Use Scale (SEAMS)	Barriers to adherence	All validated chronic conditions	High internal consistency in patients with high or low literacy	Time-consuming

Medication Adherence Report Scale (MARS)	Barriers to medication adherence Beliefs to medication adherence	Chronic mental illness, especially with schizophrenia	Simplistic scoring Strong positive correlations compared to DAI and MAQ	Limited generalizability
